# Diet of Strong Men

**Published:** 1890-02

**Authors:** 


Diet of Strong Men.—The Roman soldiers who built such wonderful roads, and carried a weight of armor and luggage that would crush the average farm hand, lived on coarse brown bread and sour wine. They were temperate in diet, and regular and constant in exercise. The Spanish peasant works every day and dances half the night, yet eats only his black bread, onion, and watermelon. The Smyrna porter eats only a little fruit, and sometimes olives, yet he walks off with his load of a hundred pounds. The Coolie, fed on rice, is more active, and can do more work than the negro fed on fat meats.—*Exchange.*

The Harvard Dental School recently received a gift of $1,000 to be added to its endowment fund.

